# Highlighting the idea of exerkines in the management of cancer patients with cachexia: novel insights and a critical review

**DOI:** 10.1186/s12885-023-11391-3

**Published:** 2023-09-20

**Authors:** Amirhossein Ahmadi Hekmatikar, André Nelson, Aaron Petersen

**Affiliations:** 1 Department of Physical Education & Sport Sciences, Faculty of Humanities, Tarbiat Modares University, Tehran, 14117-13116 Iran; 2 Institute for Health and Sport, Victoria University, Melbourne, VIC Australia

**Keywords:** Exerkines, Cancer-related cachexia, Exercise, Physical exercise

## Abstract

**Background:**

Exerkines are all peptides, metabolites, and nucleic acids released into the bloodstream during and after physical exercise. Exerkines liberated from skeletal muscle (myokines), the heart (cardiokines), liver (hepatokines), white adipose tissue (adipokines), brown adipose tissue (batokines), and neurons (neurokines) may benefit health and wellbeing. Cancer-related cachexia is a highly prevalent disorder characterized by weight loss with specific skeletal muscle and adipose tissue loss. Many studies have sought to provide exercise strategies for managing cachexia, focusing on musculoskeletal tissue changes. Therefore, understanding the responses of musculoskeletal and other tissue exerkines to acute and chronic exercise may provide novel insight and recommendations for physical training to counteract cancer-related cachexia.

**Methods:**

For the purpose of conducting this study review, we made efforts to gather relevant studies and thoroughly discuss them to create a comprehensive overview. To achieve this, we conducted searches using appropriate keywords in various databases. Studies that were deemed irrelevant to the current research, not available in English, or lacking full-text access were excluded. Nevertheless, it is important to acknowledge the limited amount of research conducted in this specific field.

**Results:**

In order to obtain a comprehensive understanding of the findings, we prioritized human studies in order to obtain results that closely align with the scope of the present study. However, in instances where human studies were limited or additional analysis was required to draw more robust conclusions, we also incorporated animal studies. Finally, 295 studies, discussed in this review.

**Conclusion:**

Our understanding of the underlying physiological mechanisms related to the significance of investigating exerkines in cancer cachexia is currently quite basic. Nonetheless, this demonstrated that resistance and aerobic exercise can contribute to the reduction and control of the disease in individuals with cancer cachexia, as well as in survivors, by inducing changes in exerkines.

## Introduction

Cancer-related cachexia (CC) is a devastating, multifactorial, and often irreversible syndrome that affects approximately 50–80% of cancer patients, depending on the type of tumor [1]. CC is a disorder characterized by weight loss with specific loss of skeletal muscle and sometimes also loss of adipose tissue [2]. Symptoms of CC include severe body mass loss, anorexia, generalized inflammation, and marked muscle wasting, leading to a severe reduction in quality of life [3]. These can also limit treatment options, as CC patients are usually less tolerant of radiotherapy and chemotherapy due to general weakness and discomfort [4]. Unlike starvation, which mainly affects adipose tissue, skeletal muscle is the main target of wasting in CC patients, suggesting a different mechanism that targets muscle loss [5]. Although the primary tissue affected by cachexia is skeletal muscle, CC cannot be reduced and eventually affects other tissues such as the liver, heart, adipose tissue, and brain [6]. Epidemiological data show that the prevalence of cachexia varies among different cancers, and overall, CC is believed to be directly responsible for up to 20% of cancer-related deaths [7]. When CC occurs is still not completely clear; however, it is known that CC can be considered one of the symptoms of metastatic cancer [8].

The effects of exercise and physical activity on health and fitness are well known [9]. Exercise is the number one priority for good health and physical fitness, but when it comes to many diseases, exercise is seen as a secondary therapeutic strategy [10]. Clinical benefits of exercise during cancer can include reducing the severity of the disease, reducing the side effects of chemotherapy, and managing negative physiological changes, and after cancer or discharge from the hospital, the return of quality of life [11, 12]. However, the molecular mechanisms underlying the beneficial effects of exercise in cancer are still poorly understood [13]. Nonetheless, it has recently been proposed that exercise-induced secretory factors, termed exerkines [14], have the potential to improve numerous aspects of health [13] and may, therefore, be involved in mediating or moderating the beneficial effects of exercise in cancer. Exerkines encompass a wide variety of signaling moieties released in response to acute and/or chronic exercise that exert their effects through endocrine, paracrine, and/or autocrine pathways [13]. These include factors released during exercise from skeletal muscle (myokines), the heart (cardiokines), liver (hepatokines), white adipose tissue (adipokines), brown adipose tissue (batokines) and neurons (neurokines) [13]. It has been suggested that the exerkine response can provide an insight into multi-organ changes following exercise [14]. In recent years, several studies related to exerkines have been published, the results of which focus on health and disease management [13, 15, 16]. Researchers responsible for these studies believe that the specificity of the exerkine responses could provide a new approach to developing therapeutic exercise recommendations targeted toward particular diseases [13, 15, 16].

**Fig. 1:**
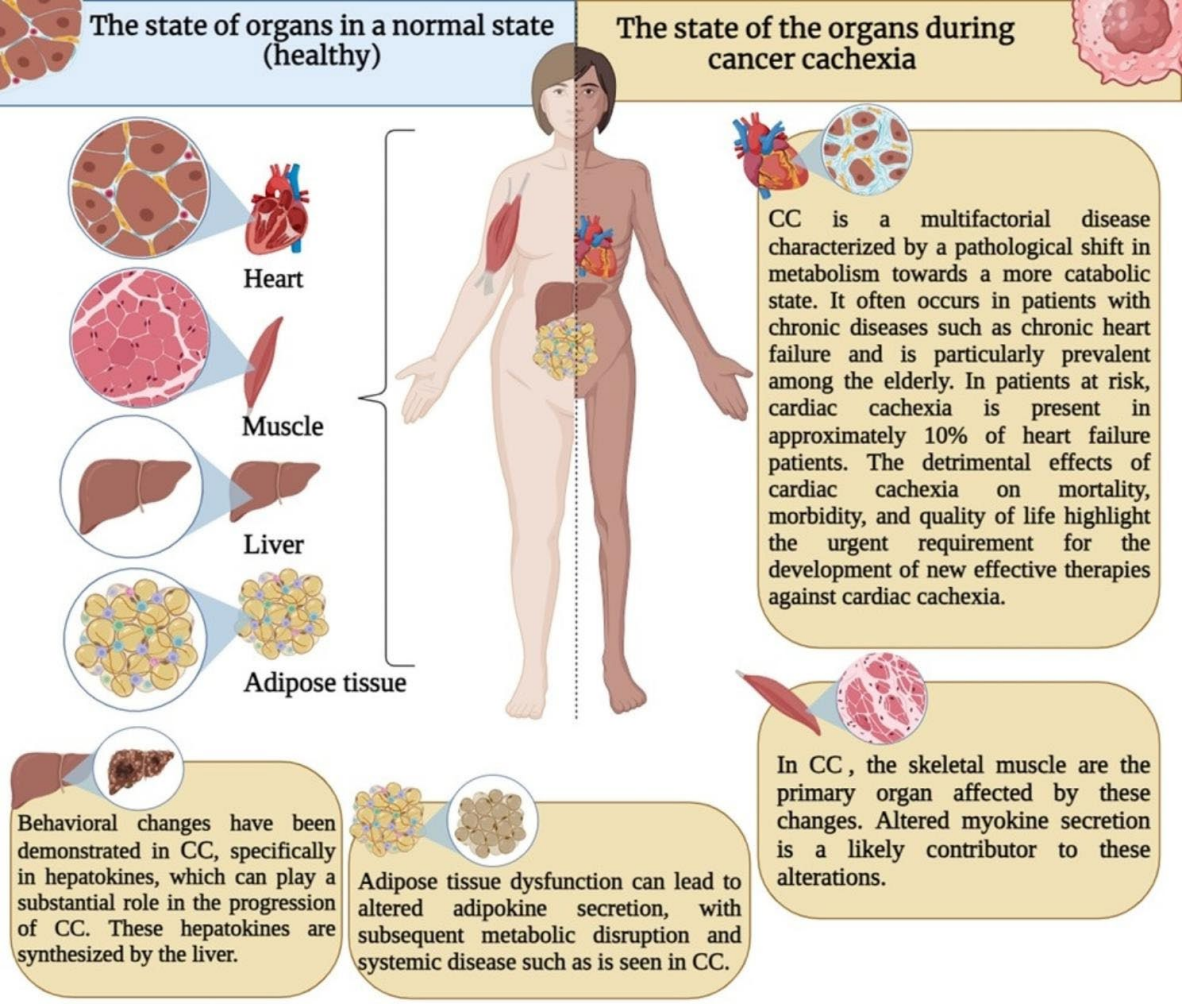
Association between CC and exerkines

In this review we intend to address the issue of whether exerkines can be effective in managing CC, with a unique look at the influence of exercise programming variables on exerkine responses; and how exerkines can lead to CC management. This novel insight could create direction for new research addressing exercise regimens targeted at beneficial exerkine responses for various clinical situations (See Fig. 1). For the purpose of conducting this study review, we made efforts to gather relevant studies and thoroughly discuss them to create a comprehensive overview. To achieve this, we conducted searches using appropriate keywords in various databases. Studies that were deemed irrelevant to the current research, not available in English, or lacking full-text access were excluded. Nevertheless, it is important to acknowledge the limited amount of research conducted in this specific field. In order to obtain a comprehensive understanding of the findings, we prioritized human studies in order to obtain results that closely align with the scope of the present study. However, in instances where human studies were limited or additional analysis was required to draw more robust conclusions, we also incorporated animal studies.

## Highlighting exerkines in cancer cachexia

### Myokines

Skeletal muscle corresponds to approximately 40% of the total body weight. This tissue secretes several factors that act in an autocrine, paracrine, and/or endocrine manner to regulate the physiology of muscles and distant organs [17]. These secretory agents were named myokines, which are synthesized and released by myocytes during muscle contractions. Secretory analysis of human myocyte culture media has identified more than 600 myokines [18]. However, most of these myokines are still not well characterized. Only a few have been studied for their biological activity and function, providing clear evidence that they are released directly from muscle contraction [18].

Epidemiological data show that skeletal muscle wasting is seen in most cancers [19, 20]. Skeletal muscles are more affected than other organs during CC [21, 22], however our understanding of why skeletal muscle is more affected by CC is incomplete. For this reason, researchers have shown an increasing interest in investigating the underlying causes of skeletal muscle wasting in cancer patients [23]. Most studies have focused their attention on muscle atrophy pathways and key proteins involved in muscle protein degradation, such as atrogin-1 and MuRF-1 [24]. It has been found that targeting myokines can be a therapeutic strategy for CC management [25]. This could indicate that, in cancer patients, skeletal muscle myokines and/or their biological effectiveness are affected by the adverse effects of CC. Thus far, only a few myokines are known to be affected by CC e.g., ↑myostatin, ↓follistatin, ↑irisin, ↑IL-6, ↑FGF-21, and possibly ↓myonectin [25–31] (Table 1).

Myostatin is a negative regulator of skeletal muscle mass, with high levels causing muscle atrophy, while low or no expression causes muscle hypertrophy [32]. Skeletal muscle atrophy and adipose tissue loss due to myostatin overexpression suggests potential for a role in the pathogenesis of CC [21]. In support, some studies report myostatin overexpression in CC [1, 27, 33]. High levels of myostatin inhibit proliferation and differentiation of satellite cells and block muscle protein synthesis [34]. It has been determined that reduction of myostatin reduces circulating inflammatory cytokine concentrations, including TNF-α and IL-6 [35] which could be useful in CC given the chronic inflammation present. On the other hand, follistatin has an opposing biological role to myostatin. Follistatin binds to and inhibits activin A and myostatin, increasing skeletal muscle hypertrophy [36, 37]. In CC, the level of follistatin is significantly reduced, and so therapeutic strategies to increase follistatin could reduce or prevent atrophy [38]. Follistatin also appears to act as an anti-inflammatory agent, reducing IL-6 [39] (Table 1).

Irisin is one of the newest myokines that many researchers are interested in investigating. Irisin may be a key molecule involved in crosstalk between muscle and fat tissue [40]. The expression of irisin in muscle is 200 times that of adipose tissue, and it has an important role in lipid metabolism by turning white adipose tissue into brown adipose tissue to increase metabolism and thermogenesis and reduce body fat percentage [41]. Given that muscle loss is often accompanied by fat loss in CC, it is of interest to investigate alterations in irisin [40]. It has been found that, in cancer patients, the increase of irisin can lead to the proliferation of cancer cells [42, 43]. However, until now, no study has been conducted that has investigated this myokine with the aim of determining an effect on cachexia, with only Altay et al. showing that irisin is increased in CC [40] (Table 1).

IL-6 is another myokine that is affected in CC. IL-6 is a cytokine with pleiotropic functions in various tissues and organs. After prolonged exercise, skeletal muscle produces and releases significant levels of IL-6, which is consequently considered a myokine [44]. In this regard, it has been found that IL-6 plays an important role in the development of CC [45]. Interleukin 6 can stimulate muscle protein synthesis, is an anti-inflammatory factor, but can also be a risk factor in CC [46–48]. These conflicting effects of IL-6 have been attributed to a temporal function based on acute versus chronic IL-6 exposure [45]. It appears that when the body experiences cachexia due to chronic inflammation, the level of IL-6 is elevated, and IL-6 can suppress protein synthesis and activate several protein degradation pathways [49, 50]. However, studies have cautiously reported that IL-6 may directly contribute to decreased protein synthesis [51, 52]. Other studies have suggested that increased IL-6 in CC may lead to suppression of IGF-1 and inhibition of AMPK activation [50, 53] (Table 1).

In CC, along with increased IL-6, FGF-21 is also increased, which appears to be a new player in the regulation of muscle mass [54]. FGF-21 is a secretory myokine that can also be released into the bloodstream by other organs, such as the liver, heart, WAT, and BAT. In skeletal muscle, FGF-21 expression is almost undetectable under healthy conditions and circulating FGF-21 is mainly produced and released by the liver [54]. In a study by Franz et al. 2019, it was found that patients with CC had the highest levels of FGF-21 compared to healthy individuals [55]. Elevated FGF-21 in CC appears to lead to the activation of muscle atrophy pathways [54]. Deletion of FGF-21 can lead to the prevention of muscle atrophy, and the overexpression of FGF-21 can lead to the induction of autophagy, and muscle loss of up to 15% [54] (Table 1).

Recently, researchers have identified myonectin as a myokine. Myonectin (CTRP15) is mainly expressed and secreted by skeletal muscle [56]. Myonectin appears to mediate cross-talk between skeletal muscle and other metabolic compartments, such as adipose tissue and the liver, to coordinate the integration of whole-body metabolism [56]. Consistent with this concept, myonectin expression and secretion by the skeletal muscle are highly responsive to acute nutritional and metabolic changes (e.g., fasting/re-feeding cycles and exercise) and chronic changes in the animal’s energy status (e.g., diet) [56]. It has been determined that myonectin is released into the bloodstream through muscle contraction. Myonectin increases the expression of fatty acid transfer genes such as CD36, FATP1, Fabp1, and Fabp4, which in turn enhances the absorption of fatty acids into cells [56–58]. It is known that autophagy levels are high in skeletal muscles of patients with CC. Autophagy is a mechanism that causes muscle atrophy [59]. Myonectin has been shown to suppress autophagy via the PI3K/Akt/mTOR signaling pathway [56–58]. ollowing mtDNA depletion, myonectin levels are significantly increased, and it enhances glucose uptake and fatty acid oxidation through activation of the AMPK signaling pathway in mouse skeletal myocytes [60, 61]. Consequently, research conducted on this emerging myokine has demonstrated that it can reduce autophagy and inflammation via the PI3K/Akt/mTOR signaling pathway [21]. This mechanism has the potential to prevent muscle atrophy in CC patients and promote increased synthesis of muscle proteins. Furthermore, it also has an impact on mitochondrial biogenesis [21] (Table 1).

### Adipokines

Adipose tissue produces pro-inflammatory and anti-inflammatory mediators that affect local and systemic inflammation. Among these mediators are adipokines, proteins produced by white adipose tissue cells that act as hormones [62]. Adipokines include leptin, adiponectin, resistin, chemerin, visfatin, omentin, vaspin, progranulin and CTRP-4 [62]. Although skeletal muscle wasting due to increased protein breakdown is recognized as the main characteristic of CC, adipose tissue depletion and remodeling is also an important clinical feature in cancer patients. Recently, adipose tissue breakdown has been shown to occur before the appearance of other classic markers of cachexia (i.e., fat loss is a more rapid event compared to muscle loss) [63, 64]. CC causes inflammation and lipid dysfunction, which leads to impaired synthesis and secretion of several pro-inflammatory and anti-inflammatory adipokines [65]. However, the role of adipokine dysregulation in cancer-induced cachexia has not yet been fully elucidated. Most of the studies on this topic are limited to adipokine changes and only relate their plasma concentrations to tumor size, while changes in synthesis, secretion and signaling in adipose tissue remodeling during CC have not yet been studied in depth [65]. Available data appears to show that only a few adipokines play a significant role in the development of cachexia in cancer (↑leptin, ↓adiponectin, and ↑resistin) [66, 67]. It has been found that leptin has the greatest effect on causing inflammation and fat loss in CC [67]. Leptin is a 16 kDa non-glycosylated protein produced by subcutaneous adipose tissue [68]. Lep-R is expressed on immune cells, and leptin mainly binds to them through the JAK-STAT and NF-kB dependent pathway in order to affect the immune response [69]. In CC, leptin increases several inflammatory factors, such as, TNF-α, IL-6 and IL-1β [70]. In confirmation of these findings, Santos-Alvarez et al. reported that leptin increases the proliferation of monocytes and causes the expression of inflammatory cytokines (TNF-α and IL-6) in the body [71]. Adiponectin has an anti-inflammatory function [72], and in cancer patients there is an observed decline in adiponectin and an increase in inflammation [73]. It has been found that mice deficient in adiponectin have increased numbers of classically activated M1 macrophages in their adipose tissue. M1 macrophages increase the production of cytokines TNF-α and IL-6 [74]. In the meantime, it has been found that the increase of adiponectin can suppress the function of eosinophils. Eosinophils are responsible for activating M1 and IL-4, which can increase inflammation [75]. It has also been found that the increase of adiponectin can be one of the main factors preventing the suppression of neutrophils [76]. Consistently, adiponectin suppresses neutrophil membrane ceramide accumulation and inhibits neutrophil apoptosis via AMPK [77]. In monocytes/macrophages, adiponectin suppresses the production of TNF-α and IL-6 and induces the production of IL-10 and IL-1 receptor antagonist anti-inflammatory mediators [78, 79]. Cancer-induced chronic inflammation has emerged as a key driver of CC, due to the multiorgan pathology and associated wide range of metabolic and endocrine disorders that lead to tissue dysfunction [65]. High levels of circulating proinflammatory cytokines, such as TNF-α and IL-6, have been observed in cachectic patients [80]. This could be due to changes in the concentration of leptin, adiponectin, and other adipokines [80]. Interestingly, increased expression of IL-6 in adipose tissue is positively correlated with increased circulating levels of IL-6. Thus, it suggests that adipose tissue, especially subcutaneous adipose tissue, may act as a key source of inflammatory mediators during the progression of CC [65]. In mouse models of CC, the secretion of pro-inflammatory cytokines by white adipose tissue, such as TNFα and IL-6, has been observed. These molecules can cause a decrease in fat storage and as a result atrophy of adipose tissue [65]. On the other hand, the deformation of fat cells and the gradual transformation of white fat cells into thermogenic fat cells (called browning) is one of the main developments of CC, which indicates high energy expenditure [81] (Table 1).

In cancer patients with CC, the amount of omentin in the blood is low [82]. Importantly, omentin suppresses monocyte adhesion to TNF-α-activated endothelial cells by inhibiting the expression of ICAM-1 and VCAM-1 via PI3K-AKT signaling by blocking the ERK/NF-κB pathway [83]. Other studies have indicated that omentin may play an anti-inflammatory and vasoprotective role in reducing obesity-related vascular complications [83, 84]. It has also been reported that omentin levels in subjects with respiratory infections are associated with the inflammatory response, and overexpression of omentin can decrease the expression of IL-6 and TNF-α and decrease the activation of the NF-κB Rel subunit [85] (Table 1).

### Cardiokines

A body of evidence shows that peptides or proteins secreted from cardiac cells could be considered cardiokines [86]. Most cardiokines, as mediators, play an essential role in maintaining the homeostasis of a healthy heart or responding to myocardial injury [86]. It has been reported that cardiokines are physiologically involved in stress response, damage repair, and myocardial regeneration and can participate in protein synthesis in end-organ tissues and systemic metabolic processes [86]. In addition, cardiokines have differential expression in response to varying physiological conditions of the heart. Secreted cardiokines are thought to maintain healthy cardiac function through paracrine/autocrine pathways or influence the response of cardiomyocytes and cardiac fibroblasts (CFs) to pathological abnormalities caused by cardiac injury or damage [86]. Natriuretic peptides, and in particular ANP and BNP, secreted by the cardiovascular system, have a particularly large impact on the occurrence and development of CVD in a paracrine/autocrine manner [87]. In addition to skeletal muscle wasting, CC is also associated with cardiac muscle dysfunction. Cardiac abnormalities are commonly seen in cancer patients and are the leading cause of death in at least one-third of cancer patients [81]. Cardiac abnormalities manifest as cardiac muscle atrophy, fibrosis, and ultimately cardiac dysfunction. This condition significantly impacts the quality of life and reduces overall survival [88]. It has been established that cancer patients commonly experience symptoms of chronic heart failure, including fatigue, shortness of breath, and decreased exercise tolerance [81]. Additionally, cardiac cachexia involves metabolic alterations, primarily including increased energy expenditure, activation of the UPR system leading to proteolysis, ubiquitin-mediated proteasome degradation, and autophagy [66, 89]. Cardiokines include atrial natriuretic peptide, brain natriuretic peptide, IL-33, IL-6, IL-18, IL-1β, follistatin, TGF-β, Ang-II, and TNF-α [90]. Limited research has examined the significance of cardiokines in CC. However, it is evident that several cardiokines undergo significant alterations in the context of CC (↓ANP, ↓BNP, ↓IL-33, ↑IL-6, ↓IL-18, ↑IL-1β, ↓follistatin, ↑TGF-β, ↓Ang-II, and ↑TNF-α) [5, 21, 91]. Follistatin and TNF-α levels have been found to increase in cardiac cachexia and can effectively increase inflammation and atrophy of the heart muscle [92] (Table 1).

### Hepatokines

Several hepatokines have been identified and investigated for their role in the development of obesity, insulin resistance, and non-alcoholic fatty liver disease [93]. However, the role of hepatokines in CC has not been investigated in detail yet. Hepatokines include Activin-E, ANGPTL3, ANGPTL4, ANGPTL6, ANGPTL8, Fetuin-A, FGF-21, Follistatin, GDF15, Hepassocin, IGF1, LECT2, Lipocalin 13, Selenoprotein-P, SMOC1 and Tsukushi [94] with several of them shown to be altered in CC (↓Activin-E, ↑ANGPTL-4, ↑FGF-21, ↓Follistatin and ↓IGF1) [95–99]. These changes can lead to increased inflammation, a negative effect on muscle myokines, a negative effect on adipokines and the development of cachexia [100–102] Table 1. Finally, it should be noted that while the importance of hepatokines in CC has not been extensively studied, the expression of these proteins can lead to the exacerbation and increase of cachexia during cancer.

**Table 1:** Changes in exerkines in CC

Changes in cachexia	Exerkines	Increase	Decrease	not clear
Skeletal muscle	**Myokines**			
	Myostatin	✓		
	Follistatin		✓	
	irisin	✓		
	IL-6	✓		
	FGF-21	✓		
	Myonectin			✓
Adipose tissue	**Adipokines**			
	Leptin	✓		
	Adiponectin		✓	
	Resistin	✓		
	Chemerin	✓		
	Visfatin		✓	
	Omentin	✓		
	Vaspin	✓		
	Progranulin			✓
Heart	**Cardiokines**			
	ANP		✓	
	BNP		✓	
	IL-33		✓	
	IL-18		✓	
	IL-6	✓		
	IL-1β	✓		
	Follistatin		✓	
	TGF-β	✓		
	Ang-II		✓	
	TNF-α	✓		
Liver	**Hepatokines**			
	Activin-E		✓	
	ANGPTL-4	✓		
	FGF-21	✓		
	Follistatin		✓	

Not clear: The available results are ambiguous or there is no information related to it.

## Acute and chronic exercise effects on exerkines

As shown in the above section, numerous kines from multiple organs and tissues are affected by and may contribute to CC. There is growing evidence that the abundance of many of these kines are favourably altered by exercise and this may be one of the mechanisms by which exercise improves health. As such, an understanding of the effects of exercise on these kines may help in the development of effective exercise interventions for treating CC and will be the focus of the following section.

### Exercise effects on muscle-derived exerkines (myokines)

There have been numerous studies examining the impact of resistance and aerobic exercise, both in acute and chronic forms, on myokines. However, there is limited research regarding the effects of exercise on myokines in cancer patients. Nonetheless, a review conducted by Kim et al. (2021) [103] revealed that alterations in myokine levels could directly inhibit cancer growth by impeding proliferation. Moreover, myokines induced by exercise play a crucial role in enhancing cytotoxicity and facilitating immune cell infiltration into tumors. In line with this, findings from other review studies validate the potential effectiveness of exercise-induced myokines in cancer [104–106]. Nevertheless, these studies did not primarily focus on investigating the impact of exercise-induced myokines on the management of CC and its survivors.

#### Responses to acute exercise

Most, but not all studies indicate that myostatin is reduced following a single bout of either aerobic, HIIT or resistance exercise. As an example, Gholamali et al. (2015) observed that cycling at 70% of VO_2_max in a single session resulted in an immediate 45% reduction in myostatin levels after exercise, followed by a further 54% decrease four hours post-exercise [107]. They proposed that one possible reason for the reduction in plasma myostatin levels following endurance activity is the concurrent increase in insulin-like growth factor-1. This increase in IGF-1 levels within skeletal muscle leads to a decrease in FoxO pathway activity, which is a crucial cellular pathway involved in promoting apoptosis [107, 108]. In line with this study, Pugh et al. (2015) showed that high-intensity interval training at 90% HRmax could decrease myostatin immediately, 2 h, and 6 h after exercise [109]. These results are consistent with other studies that reported that acute high-intensity exercise between 80 and 90% of VO_2_max or at an intensity of 90% HRmax could decrease myostatin [110–113]. In contrast to these results, Kabak et al. (2018) showed that high-intensity interval exercise (Wingate test) could increase myostatin immediately after exercise [114]. These researchers reported that the increase in myostatin was due to a decrease or lack of IGF-1 secretion and the increase of IL-6 [114]. Dalbo et al. (2011) showed in their study that three sets of 10 repetitions (80% 1RM) could decrease myostatin [115]. In line with this study, Gonzalo et al. (2013) showed that resistance exercise of three sets of 12 repetitions (80–85% 1RM) could decrease myostatin [116]. In their study, Matthew et al. 2013 investigated the effect of acute resistance exercise of 4 sets of 10 repetitions (with an intensity of 90% 1RM) on the transcriptional activity of myostatin [117]. These researchers stated in their results that acute resistance exercise reduces myostatin signaling through the activation of the TGFβ Notch inhibitor and leads to a decrease in the transcriptional activity of myostatin. The results are consistent with other studies that stated that an acute resistance training session (between three to four sets and 10 to 15 repetitions at 80 to 95% of 1RM) could reduce myostatin [118, 119]. For instance, in the study conducted by Shabkhiz et al. (2021), it was discovered that acute resistance training leads to an increase in myostatin levels when performed at an intensity of 80% of 1RM, with 10–12 repetitions and 3–4 sets [118]. It also seems that low-intensity resistance training (60% of 1RM) with blood-flow restriction can also be effective in decreasing myostatin [120]. However, in another study, it was reported that low-intensity training (40–50% of 1RM) combined with blood flow restriction could not affect the reduction of myostatin [121].

There are various reports regarding follistatin. Most studies have reported stimulation of follistatin in response to acute resistance exercise with intensity of 70–90% 1RM, 8–15 repetitions and between 3 and 4 sets [117, 122–124]. Endurance exercise also stimulates follistatin release. For example, running on a treadmill at a speed of 15–30 m/min for 35 min lead to follistatin stimulation [125]. Another study found that an acute high-intensity SIT cycle session consisting of four 30-second maximal efforts with a 4-minute recovery between sets could induce follistatin [126].

Irisin appears to be stimulated by resistance exercises with an intensity between 70 and 90% 1RM, but high-intensity exercises with an intensity of 70 to 95% VO_2_max, such as running or cycling, have a greater effect on irisin stimulation and duration [127–129]. It seems that this intensity can keep irisin levels high for 2–3 h after exercise [130–132]. Another study demonstrated that circuit training, consisting of 12–15 repetitions at 65–70% of 1RM, and performed for 3 sets, did not have any effect on irisin levels [133]. However, other studies stated that resistance training with an intensity of 70–85% 1RM and 6–12 rep can be effective in increasing irisin [134, 135].

In relation to IL-6 it seems that acute exercise, such as running or cycling at 70 to 95% of VO_2_max, can lead to an increase of IL-6 [136–141], Regarding resistance training, research has indicated that acute resistance training sessions with intensities ranging from 70 to 85% of 1RM and 6–12 repetitions can potentially lead to an increase in muscle IL-6 levels [141, 142]. However, it is important to note that the existing studies are limited and yield contradictory findings, emphasizing the need for further investigation in the future. Nonetheless, it can be cautiously suggested that acute and short-term resistance training may result in the release of IL-6. This assertion finds support in the fact that exercise intensity and mechanical stress play a role in mediating the release of IL-6 from skeletal muscle fibers. Additionally, lactate production in skeletal muscle has been proposed as a mediator of the IL-6 response to exercise [142]. On the other hand, there is evidence that an exercise-induced increase in AMPK activity in skeletal muscle correlates with IL-6 production [143]. It is worth noting that IL-6 released from skeletal muscle fibers can act in an autocrine manner, stimulating further release of IL-6 in a positive feedback loop. This could explain the observation that IL-6 activates AMPK, which, in turn, induces exocytosis of IL-6 vesicles and potentially accounts for the exponential rise in plasma IL-6 levels observed during exercise. It is important to consider that acute high-intensity resistance training, which effectively activates AMPK and increases lactate production, seems to be associated with an increase in IL-6 release from muscle [144, 145].

**Fig. 2:**
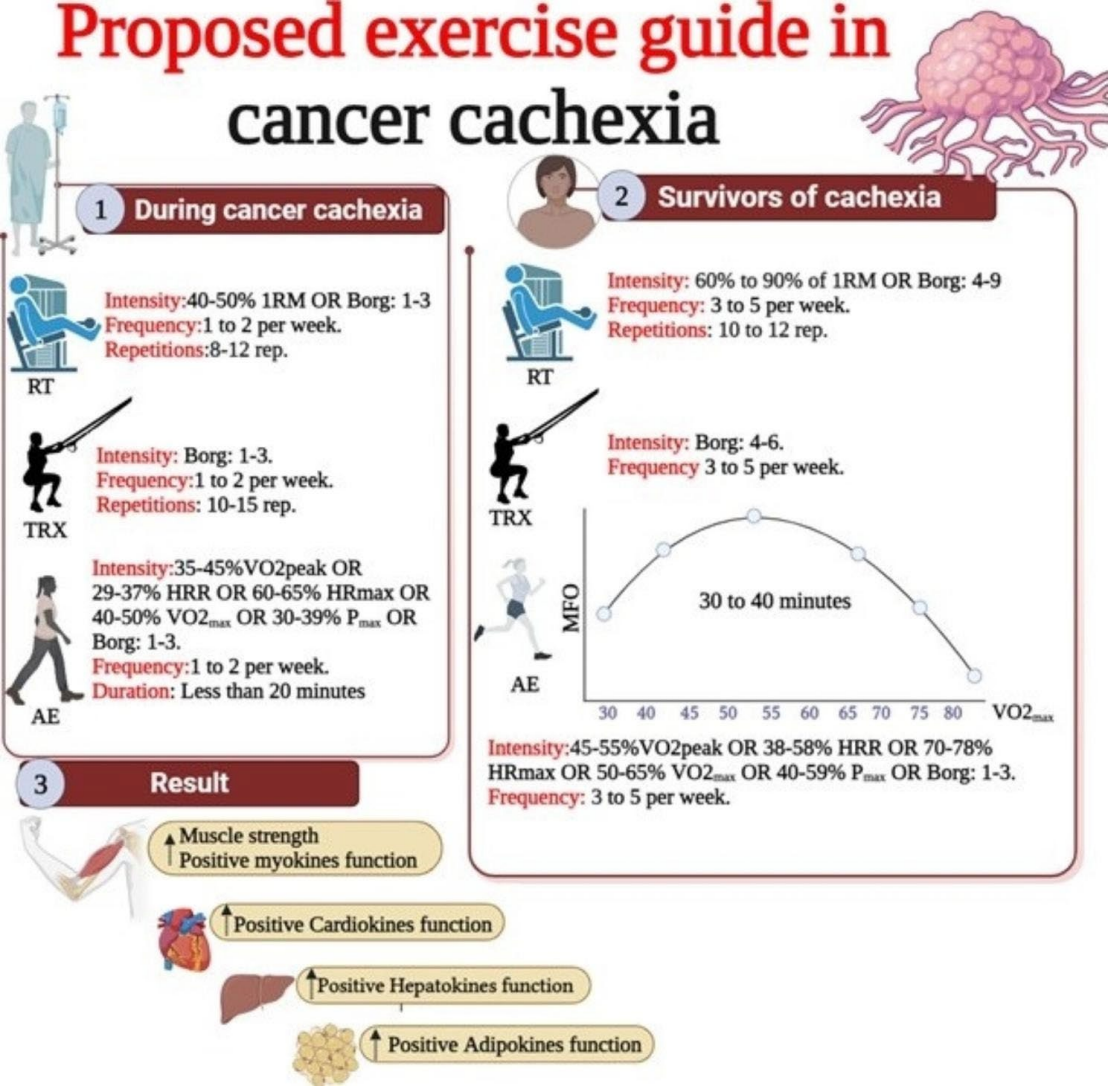
proposed exercise guide in CC. RT: Resistance training, TRX: Total Resistance Exercises, AE: Aerobic Exercise, MFO: Maximum fat oxidation

Resistance training with intensity between 70 and 90% 1RM can have a greater effect on FGF-21 between 2 and 3 h after training than cycling or running [146, 147] (Table 2; Fig. 2).

#### Responses to chronic exercise

Studies have shown that chronic exercise can significantly reduce myostatin [103, 148–151], leading to the inactivation of muscle atrophy pathways and activation of muscle hypertrophy pathways [152]. Hittel et al. (2010) reported that aerobic exercise with an intensity of 40-55% of VO_2_peak for 8 weeks could decrease myostatin levels [110]. In another study, the researchers reported that aerobic exercise with an intensity of 50–65% VO_2_max for 30–50 min for 12 weeks decreased myostatin [149]. Ko et al. (2014) showed that 30 min of running on a treadmill for 6 weeks at 45–55% VO_2_max reduced myostatin and improved muscle function [153]. In confirmation of these results, another study showed that running on a treadmill 3 times a week for 6 months was effective in reducing myostatin [154]. Aerobic training with an intensity of 50–60% of the reserve heart rate for 20–30 min continuously in the first 4 weeks and increasing the intensity to > 60% VO_2_max and up to 80% VO_2_max with a duration of 50 min can also decrease myostatin [154]. In relation to aerobic exercises, it has been discovered that engaging in these exercises at intensities ranging from a maximum of 50–65% of VO2max or 40–55% of VO2peak, with a duration of 30 to 60 min over a period of 6 to 12 weeks, can result in a reduction in myostatin levels [103, 110, 148–154].

In the study of Shahrokhian et al. (2022), it was found that resistance training can lead to a significant decrease of 15–23% in myostatin in women with breast cancer. In this study, participants performed resistance training three times per week for 12 weeks at 40-90% 1RM [155]. Furthermore, in elderly men with and without type 2 diabetes, resistance training at 70% 1RM for three sets of 10 repetitions, three times per week for 12 weeks, decreased myostatin by 5.5% (diabetic group) to 17% (non-diabetic group) relative to the non-exercising controls [118]. These findings support other studies that have also reported a decrease in myostatin levels following resistance training [112, 118, 156–160]. Chronic resistance training for 8–12 weeks and 3–4 training sessions per week, at 60-90% 1RM for 10–12 repetitions in 3 sets can be a suitable approach to reduce myostatin [112, 118, 155–160].

Six to twelve weeks of resistance training (3–5 sessions per week) at an intensity of 70–90% of 1RM between 3 and 4 sets of 10–12 repetitions can change other myokines, such as↑follistatin, ↑irisin, ↓IL-6, ↓FGF-21, and ↑myonectin [132, 156, 161–169]. Along with the reported reduction of myostatin in resistance exercise, an increase in follistatin has been seen, which can lead to activation of muscle hypertrophy [156, 158, 170, 171]. Based on the designs and outcomes of these interventions, it is evident that the intensity and duration of training play crucial roles in the observed increase in follistatin levels. Low intensity (40–50% 1RM) seems to have no effect whereas moderate (55–65% 1RM) to high intensity (70–100%1RM) can be chronically effective [156, 158, 170, 171]. Regarding irisin and FGF-21, research has indicated that resistance training can result in an increase in irisin levels and a decrease in FGF-21 levels [128, 172]. For instance, it has been found that resistance training between 6 and 26 weeks, with intensities between 60 and 95% of 1RM or an activity score of 12 to 13 on the BORG scale, can significantly increase irisin levels [173–175].

While physical activities such as Total Body Resistance Exercise (TRX) training [176–180], yoga [181–185], swimming [186–195], walking [196–208] and cycling [199, 209–212] are recommended for cachexia patients, it seems that only some of these modalities might be effective in positively changing myokines given the relative intensity involved in each.

Because of the limited results concerning myonectin in human studies, we also looked at animal studies. In animal studies it was found that exposure to three weeks of free wheel running enhanced the expression of the myonectin gene [56]. Supporting these findings, Vosadi et al. (2016) reported that moderate-intensity aerobic exercise in rats can increase myonectin levels [213]. However, there are only a limited number of studies that have reported circulating myonectin. On the other hand, it was found in human studies that eight weeks of aerobic training (50–70% of maximal heart rate for 45 min) can boost myonectin levels in women [214]. In contrast, Lim et al. (2012) found that cycling exercise at 60–80% of maximum oxygen consumption (3 sessions of 1 h per week) decreased myonectin levels [61] (Table 2; Fig. 2).

### Exercise effects on adipose tissue-derived exerkines (Adipokines)

#### Responses to acute exercise

Similar to the different responses seen in myokines due to exercise, these differences are also observed in relation to adipokines. In this regard, exercise appears to be an effective intervention for decreasing leptin, with many studies reporting a decrease in leptin after acute exercise [215–221, 205]. In general, these studies showed that high-intensity exercises of 70–95% VO_2_max and resistance exercises with an intensity of 60–80% 1RM (3 sets of 12 repetitions) can be effective in decreasing leptin by 12–26% [215–221]. Leptin levels seem to decrease after acute exercise for up to 12 h and in some studies for up to 24 h [215–221]. Also, Leal-Cerro et al., who reported that significant changes in energy expenditure may change leptin levels, have concluded that after marathon running, which caused expenditure of 2800 calories, leptin level starts to decrease [222]. On the other hand, other studies reported no leptin reduction. Dündaret al. (2019), in their study, stated that a single short-term high-intensity exercise session at 80 to 95% VO_2_max did not lead to a decrease in leptin levels [223].

The results of studies examining the effects of acute exercise on adiponectin levels are contradictory, with studies showing an increase in adiponectin or no effect on its levels [224–229]. It has been observed that both resistance training (at intensities of 70–90% 1RM, 8–15 repetitions) and aerobic training (at intensities of 70 to 95% VO2max) can lead to an increase in plasma adiponectin [224–232]. For instance, one study demonstrated that engaging in moderate and high-intensity exercises for 60 min, with intensities set at 50% and 70% of peak oxygen uptake, respectively, can lead to an elevation in adiponectin levels [230]. However, the majority of studies support the notion that acute exercise promotes an increase in adiponectin, with the intensity of the exercises being a contributing factor.

Regarding chemerin, visfatin, omentin and vaspin, the results of acute studies are limited. The currently available studies indicate that resistance exercises with an intensity of 65 to 90% (3 to 5 sets of 10 to 15 repetitions) and aerobic exercises with an intensity of 75 to 95% VO_2_max, 70 to 90% HRmax or 60 to 65% VO_2peak_ (20 to 30 min) can affect these adipokines (↓ 20–38% chemerin, ↑ 16–28% visfatin, ↓ 18.3–32.6% omentin and ↓ 6–16% vaspin) [233–243] (Table 2; Fig. 2).

#### Responses to chronic exercise

With respect to exercise type, many researchers have studied the response of adiponectin in overweight and obese humans and animals after 8–20 weeks of aerobic or resistance exercise. It seems that either aerobic exercise or resistance exercise can lead to an increase in adiponectin levels [244–247]. The results of most studies related to aerobic training indicate that 6 to 12 weeks of aerobic training with an intensity of 40 to 65% VO_2_max or 64 to 75% HRmax, for 30 to 50 min, 3 to 4 sessions per week can alter the abundance of numerous adipokines (↓leptin, ↑adiponectin, ↓chemerin, ↑visfatin, ↓omentin, ↓vaspin, ↓progranulin) [206, 207, 248–253]. In connection with resistance training, it seems that training with an intensity of 60 to 80% 1RM with 10 to 15 repetitions between 3 and 5 sets for 3 to 5 training sessions per week can also be effective in stimulating changes of these adipokines [207, 254–259] (Table and Fig. 2).

A meta-analysis of the magnitude of change in leptin levels following participation in exercise interventions lasting ≥ 2 weeks indicated that engaging in chronic exercise training is associated with a 26% decrease in leptin levels for individuals regardless of age and sex and a greater reduction in leptin occurred with a decreased percentage of body fat [260]. Also, resistance training with an intensity of 30 to 50% 1RM, 50 to 70% 1RM and 75 to 95% 1RM during a 12-month intervention could lead to a 20% decrease in leptin [199]. The authors also reported that leptin changes were strongly associated with changes in resting metabolic rate (RMR) and body mass index [261]. In contrast, 12 weeks of aerobic exercise [262], 12 weeks of resistance training [263], or 3-week combined aerobic and resistance training yielded no change in leptin levels [264]. These studies stated that the reason for the lack of effect could be due to the intensity of exercise, gender, type of measuring devices or measurement time. Finally, chronic exercise training programs have shown little effect on plasma leptin concentrations in the absence of weight loss or decreased adiposity [245, 265, 266].

Zarei et al. (2018) conducted a study to investigate the effect of eight weeks of high-intensity interval training on the serum levels of chemerin and omentin-1. In this study, rats performed HIIT five days a week for eight weeks. The results showed no significant difference between chemerin serum levels in rats undergoing an intensive exercise program compared to the control group [267]. Twelve weeks of combined aerobic and resistance exercise training, which consisted of 3 weekly sessions at an intensity of 60–70% maximum heart rate and 60–70% 1RM decreased the levels of chemerin, but did not affect the level of omentin [268]. Alternatively, Neuparth et al. (2014) evaluated, in patients with T2DM, the effect of regular moderate walking exercise (practiced for at least 30–60 min, 3–5 times a week, for a year) on chemerin. The active T2DM patients showed significantly lower levels of chemerin than those of the inactive T2DM patients [269]. Saremi et al. (2010) found that chemerin levels decreased in men performing 50 to 60 min, five days a week, of aerobic exercise for 12 weeks,, which included 15 to 50 min of walking-running, increasing weekly the exercise intensity [270]. Twelve weeks of treadmill walking and cycle ergometer exercise (5 days/week for 60 min at 85% HRmax) lead to a decrease of 18.3% of chemerin [271]. Aghapour and Farzangi (2012) reported that six weeks of running at 50% HRmax, 3 times a week, 60 min per day in obese women lead to a 26.6% reduction of chemerin [272]. Supriya et al. (2018) reported that yoga (once a week, 60 min per session for one year) in people with metabolic syndrome and high-normal blood pressure decreased chemerin [184]. Engaging in three 60-minute sessions per week for a duration of 12 weeks, consisting of rhythmic aerobic exercise performed at intensities ranging from 55 to 85% of maximum heart rate, along with core stability training, resulted in a significant reduction of 12% in chemerin levels and 18% in vaspin levels. However, there was no observed impact on plasma omentin levels [273]. A study conducted by Ribeiro Costa et al. demonstrated that an eight-week period of high-intensity interval training (HIIT) did not have any significant impact on omentin and vaspin levels in obese rats [274]. Asadi et al. (2019) compared the effect of 12 weeks aerobic exercise (70% VO_2_max), resistance exercise (11 exercises at 20% 1RM), and HIIT (six three-minute sets of running at 90% of VO_2_max) on omentin and vaspin in obese young men, finding no significant difference between the different training programmes [275] (Table 2; Fig. 2).

### Exercise effects on cardiac-derived exerkines (cardiokines)

Some studies have pointed to the importance of exercise in cardiac cachexia, and reports suggest that exercise can reduce cardiac cachexia [276–278]. Antunes et al. reported that both aerobic and resistance exercise could lead to a reduction in heart inflammation and a reduction in cardiac cachexia; however, it is unclear what exercise type (aerobic or resistance), intensity or duration are needed to affect cardiokines in cardiac cachexia [278]. Therefore, it is not possible to make a precise recommendation about what mode of exercise training and associated exercise programming variables are most suitable for stimulating cardiokines in CC and cardiac cachexia (Table 2; Fig. 2).

#### Responses to acute exercise

In patients with atrial fibrillation and healthy controls both ANP and BNP were increased at peak exercise during a graded exercise test on a cycle ergometer, although the increase was greater in atrial fibrillation patients [279]. In another study, Tanaka et al. found that plasma ANP and BNP increased in patients with hypertension and healthy controls after exercise consisting of 4 min cycling at each of 25, 50 and 75 W [87] (Table 2; Fig. 2).

#### Responses to chronic exercise

Xi et al. (2016) demonstrated that exercise increased the cross-sectional area of myocytes and the expression of follistatin. In their study, rats underwent high-intensity exercise, which involved alternating between 7 min and 25 m per minute (85–90% VO_2_max), and moderate-intensity exercise, consisting of 3 min at 15 m per minute (50–60% VO_2_max), for a total duration of 1 h. The protocol for this study involved exercising once a day, 5 days a week, for a period of 4 weeks [280].

Kamiński reported in their study that swimming at 55–75% VO_2_max for 30 min, 3 sessions per week for 6 weeks can lead to an increase in IL-6 [281]. The results of this study are in line with the results of McGinnis et al., who reported that aerobic exercise with an intensity of 60–75% VO_2_max for 30 min performed three times per week for 12 weeks can lead to an increase in IL-6 [282].

Pedersen et al. referred to IL-1β as a negative factor for the heart and reported that physical activity can limit IL-1β signaling. These researchers stated in this review that moderate intensity aerobic physical activities between 6 and 12 weeks can be a good strategy to reduce heart inflammation and IL-1β [283] (Table 2; Fig. 2).

### Exercise effects on liver-derived exerkines (hepatokines)

In this section, we will delve into the significance of exercise in relation to hepatokines. However, it is worth noting that there is a scarcity of research in this particular field. We have included animal studies alongside the human studies in order to gain a comprehensive understanding of the findings.

#### Responses to acute exercise

A study by Kersten et al. (2009) showed that an aerobic exercise intervention (2 h cycling at 50% VO_2_max) increased circulating levels of ANGPTL4 in healthy adults in the fasted but not fed state [284]. Another study found that 2 h of single-leg knee extensions at 50% of maximal workload stimulated ANGPTL4 secretion from the liver in humans [285].

In another study, the researchers stated that 2 h of aerobic training on a bicycle (intensity 60% of VO_2_ max) could lead to an increase in liver follistatin [286]. Hansen et al. (2011) reported that exercise on a bicycle ergometer with an intensity of 50% VO_2_ max lead to an increase in plasma follistatin 3 h after recovery [192].

Regarding FGF-21 changes with exercise, aerobic exercise (50% of VO_2_max for 45 min) and high-intensity interval exercise (90% of VO2max, 4 bouts of 2 min) could lead to an increase in obese mice liver FGF-21 [287]. On the other hand, Willis et al. reported in their study that moderate intensity exercise (55% peak oxygen uptake) and high intensity exercise (75% peak oxygen uptake) on 10 healthy young men could lead to an increase in plasma FGF-21 up to 4 h after training [288] (Table 2; Fig. 2).

#### Responses to chronic exercise

Catoire et al., (2014) showed that 12 weeks of endurance training had no significant effect on systemic levels of ANGPTL4 in healthy adults [289]. However, another report showed that in obese participants, 6 months of endurance training reduced body mass and increased systemic ANGPTL4 levels [290]. Nevertheless, research on chronic effects and in line with the objective of the current review was limited (Table 2; Fig. 2).

**Table 2:** A summary of exerkine responses to acute and chronic resistance training

Acute Resistance training	Exerkines	Increase	Decrease	not clear	Chronic Resistance training	Increase	Decrease	not clear
Skeletal muscle	**Myokines**				Skeletal muscle			
	Myostatin		✓				✓	
	Follistatin	✓				✓		
	Irisin	✓		✓		✓		
	IL-6	✓					✓	
	FGF-21	✓					✓	
	Myonectin			✓		✓		
Adipose tissue	**Adipokines**							
	Leptin		✓				✓	
	Adiponectin	✓				✓		
	Resistin	✓					✓	
	Chemerin	✓					✓	
	Visfatin	✓				✓		
	Omentin		✓				✓	
	Vaspin		✓				✓	
	Progranulin			✓				✓
Heart	**Cardiokines**							
	ANP			✓				✓
	BNP			✓				✓
	IL-33			✓				✓
	IL-18			✓				✓
	IL-6			✓				✓
	IL-1β			✓				✓
	Follistatin			✓				✓
	TGF-β			✓				✓
	Ang-II			✓				✓
	TNF-α			✓				✓
Liver	**Hepatokines**							
	Activin-E			✓				✓
	ANGPTL-4			✓				✓
	FGF-21			✓				✓
	Follistatin			✓				✓
	IGF-1			✓				✓
Skeletal muscle	**Myokines**				Skeletal muscle			
	Myostatin		✓				✓	
	Follistatin	✓				✓		
	irisin	✓				✓		
	IL-6	✓					✓	
	FGF-21		✓				✓	
	Myonectin			✓		✓		
Adipose tissue	**Adipokines**							
	Leptin		✓				✓	
	Adiponectin	✓				✓		
	Resistin		✓				✓	
	Chemerin		✓				✓	
	Visfatin	✓				✓		
	Omentin		✓				✓	
	Vaspin		✓				✓	
	Progranulin			✓			✓	
Heart	**Cardiokines**							
	ANP			✓				✓
	BNP			✓				✓
	IL-33			✓				✓
	IL-18			✓				✓
	IL-6	✓				✓		
	IL-1β						✓	
	Follistatin					✓		
	TGF-β							✓
	Ang-II							✓
	TNF-α							✓
Liver	**Hepatokines**							
	Activin-E			✓				✓
	ANGPTL-4	✓				✓		
	FGF-21	✓						✓
	Follistatin	✓						✓
	IGF-1			✓				✓

The available results are ambiguous or there is no information related to it.

## Measuring exerkines in studies and research gaps

The studies conducted on exercise and exerkines revealed that, in accordance with ethical guidelines for human research, blood sampling was used to measure the desired factors. However, certain exerkines, such as follistatin or interleukin 6, are derived from more than one tissue, and measuring them in blood may not yield accurate conclusions. Therefore, animal studies played a significant role in this research to determine the impact of exercise or CC on exerkines.

As always, care needs to be taken when interpreting results from animal studies as the findings may not extrapolate to humans. In Table 3, the measurement of tissues to obtain exerkine results is specified for both humans and animals. Factors marked as “Unclear” indicate variables that should be assessed through tissue analysis to obtain more accurate results. This may serve as a research gap for future studies.

**Table 3:** measurement of “kines” in CC

Organs	Exerkines	Measure Animal	Blood	Tissue	Measure Human	Blood
Skeletal muscle	**Myokines**	Skeletal muscle			Skeletal muscle	
	Myostatin		✓	✓		✓
	Follistatin			✓		Unclear
	irisin		✓	✓		✓
	IL-6			✓		Unclear
	FGF-21			✓		Unclear
	Myonectin		✓	✓		✓
Adipose tissue	**Adipokines**	Adipose tissue			Adipose tissue	
	Leptin		✓	✓		✓
	Adiponectin		✓	✓		✓
	Resistin		✓	✓		✓
	Chemerin		✓	✓		✓
	Visfatin		✓	✓		✓
	Omentin		✓	✓		✓
	Vaspin		✓	✓		✓
	Progranulin		✓	✓		✓
Heart	**Cardiokines**	Heart			Heart	
	ANP		✓	✓		✓
	BNP		✓	✓		✓
	IL-33			✓		Unclear
	IL-18			✓		Unclear
	IL-6			✓		Unclear
	IL-1β			✓		Unclear
	Follistatin			✓		Unclear
	TGF-β			✓		Unclear
	Ang-II		✓	✓		✓
	TNF-α			✓		✓
Liver	**Hepatokines**	Liver			Liver	
	Activin-E		✓	✓		✓
	ANGPTL-4		✓	✓		✓
	FGF-21		✓	✓		Unclear
	Follistatin			✓		Unclear

## Discussion and limitations of exerkine-based exercise prescription during cancer cachexia

Although exercise has demonstrated beneficial effects on various diseases and organs, our understanding of the underlying mechanisms contributing to these benefits is still limited. Additionally, the significance of exercise effects on cachexia in cancer patients and survivors has received little attention. Therefore, the objective of this study is to explore the potential role of exerkines in managing CC during and after the disease.

Currently, researchers are expanding their investigations to explore the impact of physical exercise on organs beyond skeletal muscle in CC patients. This exploration aims to establish a better understanding of the relationship and effects of these organs, ultimately leading to the development of suitable exercise protocols or strategies. Exerkines have gained increasing recognition as crucial mediators of exercise-induced changes and health benefits, particularly in terms of inter-organismic and systemic communication and coordination. However, prescribing exercise protocols for CC patients or survivors requires careful consideration due to the sensitivity of their condition. The sensitivity of prescribing exercise protocols for CC patients or survivors stems from the presence of inflammation and immunological changes in these individuals. According to Webster et al.‘s 2020 review [291], CC patients experience elevated levels of chronic inflammation, characterized by an increase in inflammatory cytokines and a decrease in anti-inflammatory cytokines. This inflammatory process is influenced by negative alterations in myokines, cardiokines, hepatokines, and adipokines. Consequently, even the slightest stress may exacerbate inflammation and contribute to the progression of cachexia. For this reason, caution should be exercised when prescribing a systemic stressor such as exercise. High-intensity exercises have been found to temporarily suppress the immune system [292, 293]. Conversely, moderate-intensity exercise appears to be a favorable strategy for maintaining or enhancing immune function [293], while low-intensity walking may have anti-inflammatory effects [294]. In this regard, a systematic review by Lavín-Pérez et al. (2023) documented that both aerobic exercise and moderate-intensity resistance exercise did not result in impaired immune system function or tumor-specific immune cell activity. Consequently, moderate-intensity resistance and aerobic exercises can be cautiously employed to enhance physiological, immunological, and psychological adaptations in cancer patients [295].

Therefore, this review aimed to comprehensively investigate the significance of exerkines in CC and its survivors, divided into two parts. The first part extensively discussed the relationship and alterations of exerkines in CC. Findings revealed that muscle (myokines), adipose tissue (adipokines), heart (cardiokines), and liver (hepatokines) are affected during CC (Figure and Table 1). These organs were identified as the primary targets of CC, leading to muscle atrophy and muscle inflammation, adverse changes in adipokines in adipose tissue resulting in chronic inflammation, negative alterations in cardiokines impacting heart function and contributing to cardiac cachexia, and hepatokines exhibiting detrimental changes in the liver that can exacerbate the negative effects of myokines and adipokines, ultimately promoting inflammation and worsening cachexia (Figure and Table 1). These negative changes not only have an impact during CC but also significantly affect the quality of life in survivors.

The second part discussed the importance of exercise on these organs. The results presented in Table 2 demonstrate that most chronic resistance and aerobic exercises have a positive effect on these organs. These exercises, characterized by chronic responses, were primarily based on moderate intensities. Based on these findings, our recommended exercise protocols are outlined in Fig. 2. Figure 2 illustrates that low-intensity exercises during cancer and moderate-intensity exercises after CC can be suitable strategies with positive effects on muscle (myokines), adipose tissue (adipokines), heart (cardiokines), and liver (hepatokines), while emphasizing caution to prevent inflammation and immune system function decline.

The present review encountered certain limitations that warrant investigation in future studies, in order to enhance the significance of conclusions pertaining to exercise, exerkines, and cachexia cancer. One of the primary limitations of this study was the scarcity of research on hepatokines and cardiokines. Therefore, it is recommended that future studies focus on investigating hepatokines and cardiokines in relation to exercise, exerkines, and the signaling pathways associated with cachexia cancer. Additionally, the current study revealed measurement limitations for certain variables, as indicated by the “Unclear” marking in Table 3. This suggests that in human studies, some variables are explained based on data derived from multiple organs, which complicates the drawing of definitive conclusions. To overcome this challenge, it is suggested that future studies in animal models, specifically those investigating exercise and exerkines in CC, measure these variables directly from tissue samples. Lastly, another limitation of the study was the focus on variables with the greatest impact on CC. To further emphasize the role of exerkines, it is recommended that future studies explore additional variables, such as GDF15. By addressing these limitations in future research, a more comprehensive understanding of the relationship between exercise, exerkines, and cachexia cancer can be achieved, leading to more prominent findings and insights.

Considering the significant knowledge gap surrounding exerkines in cancer patients, it is highly recommended that future studies concentrate on investigating the role of exerkines in cachexia cancer (CC) and their impact on various organs. These studies should also delve into the underlying immunological and physiological mechanisms involved.

In summary, exerkines present a promising avenue for future research endeavors. They hold immense potential as biomarkers for predicting outcomes and facilitating the development of personalized exercise programs aimed at improving overall health, mitigating disease, and promoting resilience across all stages of life. Furthermore, exploring the connection between exerkines and cancer could lead to a substantial breakthrough in the field of exercise oncology.

## Data Availability

All data generated or analysed during this study are included in this published article.

## Abbreviations

AMPK: AMP-activated protein kinase
Ang-II: Angiotensin II
ANGPTL4: Angiopoietin-like 4
AKT: Protein kinase B
ANP: Atrial natriuretic peptide
BAT: Brown adipose tissue
BDT: Brown adipose tissue
BNP: Brain natriuretic peptide
CC: Cancer-related cachexia
CTRP15: C1q/TNF-related protein 15
CTRP-4: C1q/TNF-related protein 4
CFs: Cardiomyocytes and cardiac fibroblasts
FGF-21: Fibroblast growth factor 21
HIIT: High intensity interval training
HRmax: Maximum heart rate
NF-Κb: Nuclear factor kappa-light-chain-enhancer of activated B cells
IGF-1: Insulin-like Growth Factor-1
IL-33: Interleukin 33
IL-6: Interleukin 6
IL-18: Interleukin 18
IL-1β: Interleukin 1β
ICAM-1: Intercellular Adhesion Molecule 1
Lep-R: Leptin receptor
MuRF-1: Muscle RING-finger protein-1
MTOR: Mammalian target of rapamycin
NF-Kb: Nuclear factor kappa-light-chain-enhancer of activated B cells
SIT: Sprint interval training
TGF-β: Transforming growth factor beta
TNF-α: Tumor necrosis factor
UPR: Unfolded protein response
VO_2_max: Maximal oxygen consumption
VCAM-1: Vascular cell adhesion protein 1
WAT: White adipose tissue
1RM: One-repetition maximum
